# Prof. S. B. Lakamanahalli[Aff AF0001]

**Published:** 2010

**Affiliations:** Professor of Anaesthesiology

**Figure:**
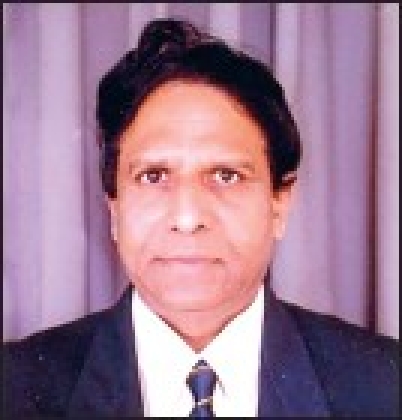
5^th^ October 1944 – 2^nd^ April 2010

Dr. Sangappa Basappa Lakamanahalli from Dharwad Dist, got his MBBS Degree and MD Anaesthesiology from KMC Hubli in 1974. He worked as Registrar in JJMMC Davangere from 03-08-1978 to 15-04-1985, Later as Assistant Professor 6 years until 01-08-1991. He was very popular teacher among the students and his own colleagues, for his simple and dignified personality. He joined JSSMC Mysore as Associate Professor in Sept. 1991, in 1992 as full time Professor, Prof. & HOD Anaesthesiology from 15-07-1996.

On Nov. 6^th^ 2006 he joined as a Senior Professor at Sri Devaraj Urs Medical college, Kolar and he was liked by one and all during his tenure. He was a unique personality, cool headed and a very kind hearted man. His honesty and simplicity are inspiration for the budding doctors. His main motto was talk less and work more. He was an excellent teacher and also had a keen interest in academic activities.

We all mourn for his absence, unexpected death, but his achievements and deeds made his presence immortal in our heart. We all pray the almighty to rest his soul in peace.

